# An Analytical Mini-Review on the Compression Strength of Rubberized Concrete as a Function of the Amount of Recycled Tires Crumb Rubber

**DOI:** 10.3390/ma13051234

**Published:** 2020-03-09

**Authors:** Luca Lavagna, Roberto Nisticò, Matteo Sarasso, Matteo Pavese

**Affiliations:** Polytechnic of Torino, Department of Applied Science and Technology DISAT, C.so Duca degli Abruzzi 24, 10129 Torino, Italy; matteo.sarasso@studenti.polito.it (M.S.); matteo.pavese@polito.it (M.P.)

**Keywords:** concrete, composite materials, rubber concrete, mechanical properties, recycling, rubber

## Abstract

Since waste tires constitute a serious environmental concern, several studies are devoted to the use of finely divided recycled rubber for the production of rubberized concrete by partial substitution of the mineral aggregate fraction. The introduction of rubber into concrete presents several advantages (e.g., improvement of toughness and thermal/electrical/acoustic insulation capacities). Unfortunately, the addition of a high content of rubber into concrete causes an important loss of mechanical resistance of the final composite. In this context, several scientific studies are devoted to investigate the best technical solutions for favoring the interfacial adhesion between rubber and cement paste, but the interpretation of the literature is often misleading. To overcome this issue, the metadata extrapolated from the single scientific works were critically re-analyzed, forming reference diagrams where the variability fields of the different rubber concrete formulations (in terms of mechanical responses as a function of the rubber content) were defined and the best performances discussed. This study evidenced the twofold role of reference diagrams, able in both presenting the data in an unambiguous manner (for a successful comparison) and providing the guidelines for future works in this research field.

## 1. Introduction

Along with human activities, the solid waste increment in landfills opened a Pandora’s box related to the end-of-life waste management, prompting worldwide researchers to identify the best technologies for favoring waste disposal and (if possible) their recycling, aiming at environmental and economic sustainability [1,2,3,4,5,6,7]. Nowadays, significant effort has been directed toward the recovery and reuse of waste materials as new resources (based on green chemistry and circular economy guidelines) for the production of value-added products [8,9,10,11,12,13,14,15]. Among the different waste types, end-of-life waste rubber tires are one of the most voluminous wastes in terms of both shape and quantity (the European consumption of tires is about 3.2 million tons per year) [16,17]. From the chemical viewpoint, the composition of tires is mainly organic (rubber/elastomers and carbon black about 70%), with a small amount of inorganic components (about 17%), textiles and other additives (about 13%) [18]. Used tires can be either reused or wasted, and end-of-life tire waste can be either landfilled, thermally converted to produce energy (via combustion) or re-processed to obtain raw materials [19]. Regarding the waste tires management, the first two options (namely, landfilling and burning) can cause several environmental concerns (e.g., serving as habitat for the proliferation of disease-carrying insects and rodents during landfilling, or being a source of dioxins and other volatile pollutants during combustion) with serious risk to human health [20,21]. Thus, for all these reasons, rubber tires are preferentially treated via milling to produce granulates, chips, powders and textiles, thus making them exploitable in several advanced applications [19]. 

In this context, the possibility of partially substituting the mineral aggregate (sand and gravel) fraction in concrete with recycled tires crumb rubber is a technical solution that is extremely appealing, as it leads to cement-based composite materials that can have significantly improved ductility, tenacity, impact resistance and thermal/electrical/acoustic insulation capacities [22]. Additionally, the development of rubber-containing concrete allows to reach a twofold environmental benefit, since it reduces both the environmental impact due to tire disposal (landfilling and burning) and the excavation processes for recovering the mineral aggregate fraction from quarries or along rivers courses.

Despite the many benefits given by the introduction of rubber in concrete, a significant reduction of both the mechanical resistance and specific mass has been registered. These side-effects are attributable not only to the different mechanical response of the rubber component compared to the mineral aggregates, but mainly to the poor interfacial adhesion between the rubber element with the cement paste [23,24], which significantly reduces the loadable quantity of rubber in concrete.

Nevertheless, promising mechanical properties (mostly the increased toughness) encouraged the evaluation of structural performances of rubber concrete where high resistance to dynamic stress is required, such as in the case of earthquake-resistant structures [25]. However, in order to make feasible the structural uses of rubber concrete, it is necessary to select formulations able to guarantee an adequate level of mechanical resistance, thus overcoming the interfacial adhesion concerns. A very recent review by Roychand and co-workers presents the current panorama of the research on the substitution of aggregates with recycled rubber [26], together with the possibilities envisaged in the literature of rubber treatment or fiber insertion to guarantee acceptable mechanical properties to the cement-based composite. 

In the current literature, rubber concrete is presented with very different formulations that are sometimes improperly compared. In fact, the literature suggests that typical parameters affecting the mechanical response of rubber concrete are (i) the rubber content (expressed either as aggregate substitution level or as volume/weight percentage), (ii) the granulometry of both the substituted mineral aggregate (coarse or fine) and the added rubber, (iii) the introduction of additives (e.g., pozzolana, silica) and (iv) the quantities of all components within the formulation and the water-to-cement ratio, (v) the effects of appropriate pre-treatments (e.g., NaOH washing) and surface functionalization (or coatings) of the rubber to favor the interface interaction with the inorganic matrix (cement paste).

In this study we aim at improving the ease of comparison between the different procedures, formulations and strategies that are currently employed to improve the properties of rubber-containing concrete. We provide a rational evaluation of the experimental results reported in the literature, first by building a reference diagram that shows the variability of mechanical response of different rubber concrete formulations. Then, we evidence the effect of different approaches used in the published literature on the final properties of rubber concrete. The final goal is to provide a preliminary assessment tool (to be adopted in future research) that allows a rapid estimation of the mechanical behavior of the produced material relative to the performance of current state-of-the-art tools.

## 2. Analysis of Bibliography

Some works focused on the use of end-of-life tires have been selected from electronic databases (i.e., Scopus, Science Direct), others from the recent review by Roychand et al. [26].

A list of studies focused on the mechanical properties of different rubber concrete formulations was produced. From the initial list, 46 publications [19,23,24,27,28,29,30,31,32,33,34,35,36,37,38,39,40,41,42,43,44,45,46,47,48,49,50,51,52,53,54,55,56,57,58,59,60,61,62,63,64,65,66,67] were selected since they followed both criteria of inclusion adopted in this research, namely (i) tests should be performed on different mixtures of rubber concrete and compared with a similar concrete composition without rubber; and (ii) numerical data of the mechanical (mostly, flexural and compression) strength should be provided.

The selected case studies were further analyzed in depth by verifying the effective relevance of their content with the aim of this study. Following this exclusion principle, 11 publications [28,29,30,31,32,33,35,36,38,39,40] were not included in this research as follows: Naito et al. and Kashani et al. [28,30] did not indicate the type of cement and aggregate used in their study. Kaloush et al. [29], Ismail et al. [31] and Raffoul et al. [35] did not keep constant the mix design when adding the rubber to concrete, so that the comparison with the reference material is not possible. Kaloush et al. and Raffoul et al. changed the w/c ratio, while Ismail et al. added polymer fibers. Xue et al and Najim et al. [32,33] did mechanical characterization for other purposes. Mendis et al. [36] and Elghazouli et al. [40] did not make compression tests. Aslani et al. [38] and Wang et al. [39] used iron or steel fibers to enhance the mechanical performance. Whereas six publications [19,23,34,41,42,43] were not included for other specific reasons, namely the study by Rahman et al. [41] was excluded since it focused on the effect of different plasticizers, the one by He et al. [23] because it is mainly focused on the adhesion phenomena occurring between concrete and rubber, the two studies by Najim et al. [19,42] together with the one by Siddique et al. [43] and Roychand et al. [26] since they are review articles, and lastly the one written by Taha et al. [34] because it presents issues related to the mix design and the relative composition quantification.

After this preliminary screening, the publications effectively analyzed in this study were 27. Since, in some cases, more than one series of samples per publication has been considered, detailed acronyms have been adopted to unequivocally recognize a specific data series in the reference diagrams. To simplify the comprehension (and the readability) of the diagrams, Table 1 reports the acronyms adopted in the present study and the corresponding description from the original study. 

## 3. Bibliography Data Manipulation

The main relevant mechanical test for evaluating the mechanical response of concrete is the evaluation of the compression strength. All the case studies considered present compression strength results, with most following ASTM C39/C39M [68] as the reference standard, while a few do not report the standard adopted for the measurement. 

The first step in the analysis was the extraction of numerical values from the compression tests of each publication. In some cases, such values were not directly reported in the text, but graphically represented in a diagram. To extrapolate the numerical values from plots and graphics, the software WebPlotDigitizer 3.8 (Austin, Texas, USA) has been employed. 

**Figure 1:**
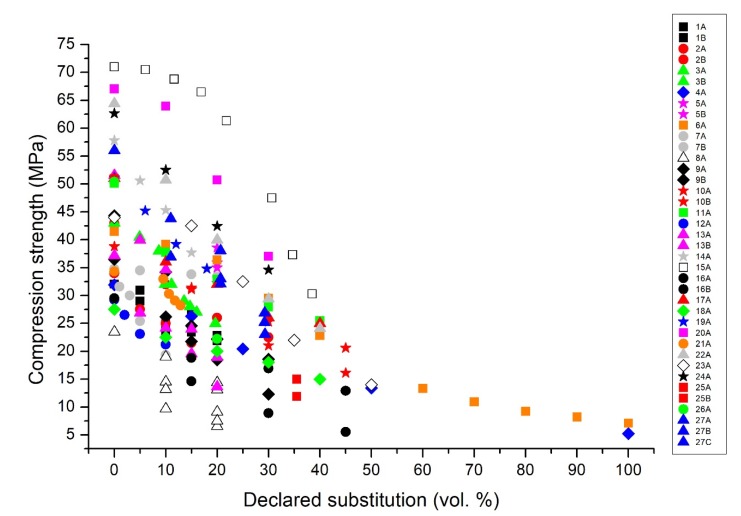
Reference diagram: compression strength (expressed in MPa) vs. the declared volumetric percentage of substitution of the mineral aggregate with rubber (expressed in vol.%).

The data extracted from the selected publications were plotted in Figure 1, which reports the compression strength declared by the authors against the declared volumetric percentage of substitution of the mineral aggregate fraction with rubber. As expected, Figure 1 reports a significant reduction of the mechanical resistance of the materials when the rubber content within the composites increases. An objective comparison of the performance of the different rubber concrete formulations is, however, hampered by two factors: first, the control concrete presents very different compression strength values, which are comprised in the very wide 22-72 MPa range. Second, most papers have different ways of declaring the substitution of the mineral aggregate fraction with rubber. Thus, both the *x*-axis and the *y*-axis do not present comparable data.

Considering the compression strength, it is evident in Figure 1 that, for instance, the values at 10% rubber substitution that are in the 50–70 MPa range (series 15A, 20A, 22A, 24A [59,63,65,67]) are very high mostly due to the use of high-strength concrete. Other series, for instance 7A and 7B [50], have a much lower reduction in strength, but since their reference concrete has a lower strength, they seem less performing with strength values in the 20–35 MPa range. The same issue happens at all percentages of rubber substitution. For instance, at 30% declared substitution, the series 20A [63] or 24A [67] seem more performing than the series 18A [61], even if the latter has a much lower strength reduction with respect to the control concrete.

The effect of the control concrete value is very evident, but also the differences in the declared substitutions have a profound effect on the lack of clarity of Figure 1. For instance, series 6A [49] declares up to 100% rubber substitution, but this substitution refers to a specific fraction of aggregates and not to the total aggregate content. This effect expands the curve toward very high values, suggesting a smaller strength reduction than the real one.

The revision of the stated formulation pointed out a significant inconsistency among the analyzed papers in the mode to declare the reference quantities. Hence, all the declared formulations were analyzed case-by-case in order to obtain the principal components of the mix design and the characterizing parameters (i.e., quantity, size, density). In most cases, in fact, the percentage of substitution did not refer unequivocally to the total volume of the aggregates, but it preferentially refers to a type (or portion) of aggregate (e.g., either coarse or fine). To guarantee uniformity in the data interpretation, the substitution level in the composites was “normalized” by referring always to the total volume of aggregates present in the control mixture.

Additionally, the vis-a-vis revision of the selected publications revealed a further remarkable inconsistency that affected the data interpretation. In fact, some authors chose the weight parameters as either specific weight or bulk density. Moreover, the choice of these parameters for every formulation was different case-by-case, often showing inconsistent values, thus making the numerical values here extrapolated extremely wide and difficult to compare. For instance, Youssf et al. [65], series 22A, declared a specific gravity for crumb rubber equal to 0.85, which is outside the values observed in the literature for vulcanized rubber. As another example, Gesoğlu et al. [51], series 8A, use tire chips with specific gravity 1.02 and crumb rubber with specific gravity of 0.83 and 0.48. It seems to us that also these values are too low with respect to the literature data of rubber density [69,70,71,72,73,74,75,76,77]. It is difficult to hypothesize the origin of these values, but it could be possible that some values are apparent density or bulk density. Other cases are those of Boudaud et al. [45], series 2A/B, who did not mention the rubber density, and of Feng et al. [54], series 11A, who cited only the bulk density of 539 kg/m^3^, which cannot be used to quantify the volume substitution.

To overcome this issue, the composition of the formulations that were not clearly defined or outside a reasonable range was further recalculated by using, for the specific weight, the average value of all the studies where the data were clear and consistent with literature values. These weight parameters are reported in Table 2. If the weight parameters provided by the original authors were comparable with the ones selected in Table 2, the declared values were maintained in our data elaboration.

Therefore, the aggregate content and the relative volumetric percentage of substitution with rubber were recalculated for all compositions using, when needed, the reference values reported in Table 2. In this way, rubber concrete mixtures considered in this study present the highest level of comparability in terms of parameters and mechanical performances registered. 

Despite this, it is important to notice that the analyzed formulations still show a considerable variability of the components used, namely (i) type of cement paste and aggregates adopted, (ii) presence/absence of additives, fly ashes, slags, silica and other additional components and (iii) amount and proportion of each component within the concrete formulation. For all these reasons, even after this consistent data homogenization, resulting case studies still present a significant degree of heterogeneity. However, at the end of this elaboration process, all case studies are coherent among each other. 

**Figure 2:**
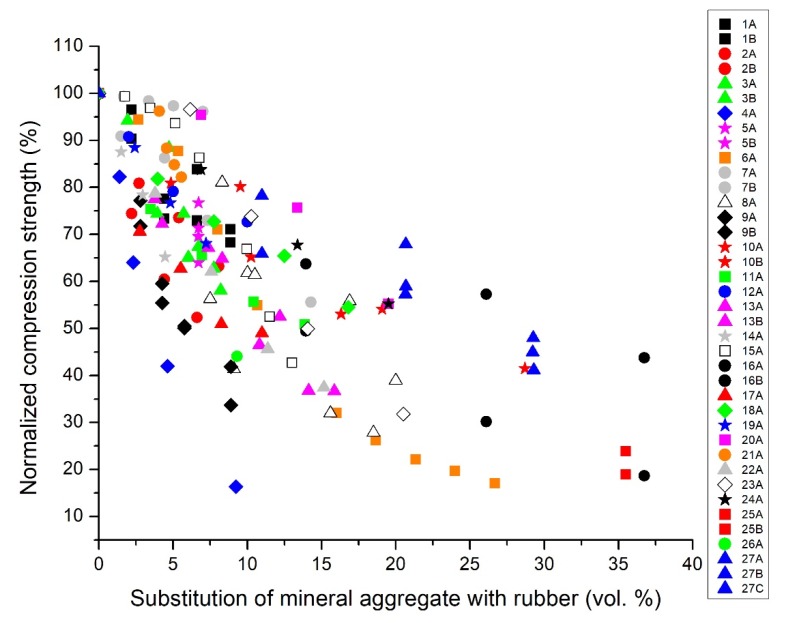
Reference diagram: normalized compression strength (expressed in %) vs. the volumetric percentage of substitution of the mineral aggregate with rubber (expressed in vol.%) recalculated using the fixed values of reference specific weight.

Figure 2 reports the recalculated compression strength, normalized with respect to the control samples, against the volumetric percentage of substitution of the mineral aggregate fraction with rubber. It is interesting to note that the maximum substitution shown on the *y*-axis becomes 37% and is no longer 100% as in Figure 1. Moreover, all series start at 100% for the reference concretes without rubber.

By using the diagram presented in Figure 2 it is possible to compare the different rubber concretes proposed by the different authors, without the risk of being misled by the different ways of presenting the experimental data. For instance, it is evident that series 7A/B, 20A, and 23A present good results at low rubber substitution, and series 27A/B/C and 16A/B at high rubber substitution; while series 4A and 9A/B lose strength rapidly with the increase of rubber content. To discriminate which parameters have a significant effect on the compression strength of rubber concrete, a complete analysis of the outputs coming from the reference diagrams obtained after this important elaboration process is reported in the following paragraphs.

## 4. Reference Diagrams and Their Critical Interpretation

The reference diagram relative to compression tests (Figure 2) clearly shows a reduction of the compression strength by increasing the rubber content (as expected). Moreover, going more in detail, it is possible to highlight different trends. Liu et al. 7B [50] obtained a reduction of only 4% of the compression strength with a substitution of the aggregate fraction with rubber of 7 vol.%., whereas other studies reported at least 15% reduction for the same amount of rubber (1AB, 3AB, 5AB, 6A, 8A, 11A, 12A, 13AB [44,46,48,49,51,54,55,56]). The works written by Zheng et al., 10AB, [53] and Najim et al., 27ABC, [57] also report interesting performances, with a lower reduction of compression strength than most of the case studies here analyzed (moreover, this difference is more evident when increasing the amount of rubber inside the composites). Conversely, the formulations proposed by Issa et al., 4A, [47] and Mohammed et al., 9AB, [52] are characterized by poor mechanical responses (below the other case studies considered) with a remarkable depletion of the compression strength with just 15 vol.% rubber substitution.

In general, the data described in Figure 2 show a mean compression strength reduction of around 20% for substitution of ca. 5 vol.%, around 40% for ca. 10 vol.%, and around 60% for ca. 20 vol.%.

**Figure 3:**
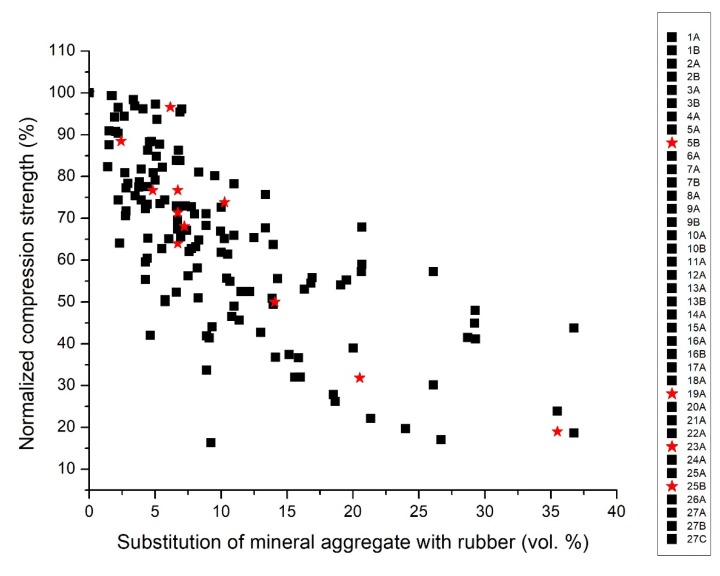
Reference diagram: normalized compression strength (expressed in %) vs. the volumetric percentage of substitution of the mineral aggregate with rubber (expressed in vol.%) recalculated using the fixed values of reference specific weight. Effect of the rubber pre-treated in NaOH (red stars) and rubber without any treatment in NaOH (black squares).

In order to rationalize these trends, the diagrams were further analyzed, studying specific aspects of the formulations reported in the literature that could have beneficial or detrimental effects on the mechanical properties of the rubber concrete. Figure 3 reports the same data of Figure 2, but highlighting the effects due to rubber pre-treatments/modifications. The surface modification of the crumb rubber by means of appropriate pre-treatments (e.g., NaOH washing, controlled oxidation) and/or further surface functionalization (e.g., coatings deposition) is a technical solution investigated by worldwide researchers to overcome the interfacial adhesion concerns with the other inorganic components (mostly the cement paste), principally responsible for the loss of mechanical properties in rubberized concrete [48]. Few data are available; however, four series (5B, 29A, 23A and 25B) can be separated from the others, since the rubber in these cases underwent a chemical treatment with NaOH. This treatment, however, showed no significant effect on the mechanical performances of the composites: the normalized compression strength values experimentally obtained are widely distributed and mainly in the central part of the measured range.

**Figure 4:**
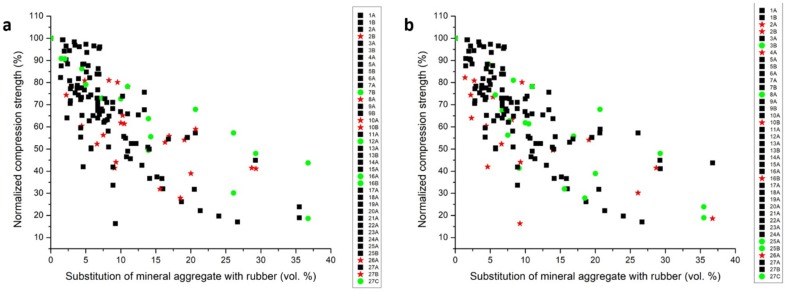
Reference diagram: normalized compression strength (expressed in %) vs. the volumetric percentage of substitution of the mineral aggregate with rubber (expressed in vol.%) recalculated using the fixed values of reference specific weight. (**a**) Effect of replacing fine aggregate (black squares), replacing coarse aggregate (red stars), replacing both fine and coarse aggregate (green circles). (**b**) Effect of using for aggregate replacement fine rubber (black squares), coarse rubber (red stars), fine and coarse rubber (green circles).

Another important parameter that could affect the rubber concrete performance is the type of aggregate that is substituted—fine, coarse, or both—and it is reported in Figure 4a, where black squares represent substitution of fine aggregate, red stars substitution of coarse aggregate, and green circles both fine and coarse aggregate. In this case, the data are again widely distributed, suggesting the absence of very significant trends of improvement or deterioration of the mechanical properties depending on the substituted aggregate, but at high rubber content it seems that the substitution of both fine and coarse aggregate entails a lower reduction of mechanical properties.

Figure 4b shows instead the effect of the replacement of the aggregate according to the size of the rubber used to replace it. From the figure it seems that using coarse rubber is less effective than using fine rubber or a mixture of fine and coarse rubber. 

However, it is very interesting to study in bigger detail the effect of the substitution of the different size fractions on the mechanical properties. In most cases, the coarse aggregate was replaced with coarse rubber and the fine aggregate with fine rubber, but there are cases where different sizes were used for the substitution. In the 2A [45] and in the 4A [47] series, the fine aggregate was replaced with large size rubber, leading to a drastic decrease in the mechanical strength. Similarly, when a smaller aggregate fraction was substituted with a larger rubber one, the mechanical results were generally not very good (series 3B [46], 25A and 25B [24], 16B [27]). On the contrary, when a bigger aggregate fraction was substituted with smaller rubber one, the mechanical results were from average to very good. An average performance was observed in the case of series 7B [50], 10A [53] and 12A [55], while a very good one in the case of series 8A [51], 16A [27] and 27B [57].

**Figure 5:**
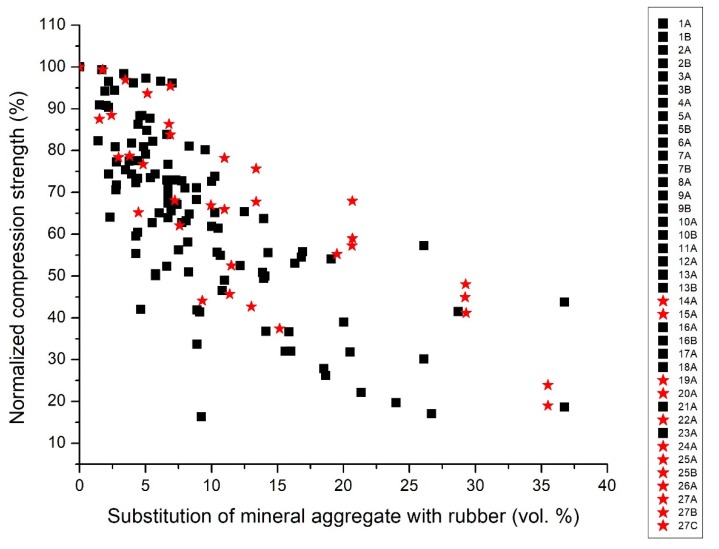
Reference diagram: normalized compression strength (expressed in %) vs. the volumetric percentage of substitution of the mineral aggregate with rubber (expressed in vol.%) recalculated using the fixed values of reference specific weight. Comparison using high-performance concrete (red stars) vs normal cement (black squares).

In Figure 5 the comparison of ordinary Portland cement (black squares) vs high-performance cement (red squares) is presented. The use of a high-performance cement seems to improve the mechanical performance of the composite, both at low and at high aggregate replacement volumes.

## 5. Considerations on Specific Case Studies

In order to rationally unveil the reason why some particular case studies present better mechanical responses than the others, here we reported a brief technical analysis of the best formulations selected. Additionally, on the basis of the reference diagrams proposed in this study, a critical analysis of the formulations and of the boundary conditions has been reported, with the aim of better understanding and proposing new guidelines for favoring the development of high-performance rubber concrete.

Among the different case studies here analyzed, the three series proposed by Najim et al. [57] and Topçu [27] (indicated in this study as 27A, 27B, 27C, 16A and 16B) seem to give the best performances at higher substitution of rubber. For the study of Najim et al., the authors selected a high-strength cement as cementitious matrix, using only a single fraction of rubber with sizes ranging between 2 and 14 mm. One hint adopted by the authors is to favor the dispersion components by premixing the solid ingredients in absence of water. As highlighted in Figure 4a, the best results were reached when both fine and coarse aggregates were replaced with rubber. Topçu [27] suggests using fine rubber for substituting both fine and coarse aggregate. Moreover, in his work it is evident that rubberized concretes in contrast to the normal ones have higher plastic energy capacities. 

The results obtained by the series from Zheng et al. [53] (indicated in this study as 10B) seems also very promising, since a lower resistance cement has been selected as cementitious matrix. However, in this case the best results were reached by replacing only the coarse aggregate fraction. Compared to the previous procedure adopted by Najim et al., 27ABC [57], the procedure employed by Zheng and co-workers 10AB [53] for the specimen preparation has not been fully described; thus, it is impossible to point out any further hints to follow. On the other hand, the only element in common between these two studies seems to be the size of the rubber adopted for high level of substitution that is around 5-14 mm size, which confirms the trend suggested by Topçu 16AB [27].

When considering the substitution of only small quantities of mineral aggregate, the best performances were reached by Liu et al. [50] (indicated in this study as 7A). As highlighted in this study, authors selected rubber powders with a small size distribution (i.e., 2–4 mm), finding more advantageous to substitute only the fine aggregate fraction. Interestingly, in this study Liu and co-authors pointed out that the use of rubber already used in the construction field allows to obtain a significant improvement of the adhesion between rubber and the cementitious paste. 

It is also evident that the use of a high-performance cement helps to reduce the loss of mechanical properties as shown in Figure 5, in particular with high percentages of aggregate substitution.

Given the graphs of reference, some points are evident to obtain good mechanical results for high percentages of substitution of the aggregate.

(i) It seems that the substitution of the aggregate with a larger fraction of rubber is not effective [45,47], while good results were obtained with rubber with a smaller size with respect to the aggregate [27,57]. The use of coarse rubber seems less effective than the use of small rubber, as shown in Figure 4.
(ii) Prior to proceeding with the addition of water, the cement paste, the mineral aggregates and rubbers should be premixed to favor the homogeneous dispersion of the components within the final composite [50].
(iii) As pointed out by He et al., Liu et al. and Sgobba et al. [23,24,50], the adhesion problems between the components can be partially overcome by imposing a light chemical treatment on the rubber, before introducing it into the desired cement paste. However, it is worth noting that, based on data analysis, the chemical treatment of rubber with chemical agents (e.g., NaOH) has almost no tangible effects on the mechanical performance of the cement composite even when using high-performance cement [24,48,62,66].
(iv) Lastly, using a high-performance cement as matrix can significantly reduce the loss of mechanical properties for high substitution degree of the mineral aggregate fraction [24,37,57,58,59,62,63,65,67].

## 6. Conclusions

Quite recently, several efforts were realized for trying to minimize the anthropogenic impact on the natural environment, and promising results were obtained in term of sustainability. Among the different classes of human-derived technological wastes, end-of-life rubber tires caught the attention of worldwide experts since, from one side, they are potentially dangerous for both environment and human health if normally landfilled or thermally converted, whereas from the other side they can be extremely appealing if re-processed to produce useful products. In this context, the possibility of partially substituting the mineral aggregate fraction in concrete with recycled rubber tires to produce cement-based composite materials with significantly improved toughness, impact resistance, and thermal/electrical/acoustic insulation capacities is an exceptionally promising solution. Unfortunately, this solution presents some technical limitations, such as the decline of the composite mechanical resistance at high content of rubber.

On the basis of the most recent results reported in the literature, we reported here a literature survey coupled with a critical analysis of the metadata extrapolated from scientific papers. Reference diagrams showing the variability fields of the different rubber concrete formulations in terms of mechanical responses as a function of the rubber content were presented and best performances (in terms of both mechanical resistance and loading of rubber) critically discussed. This study evidenced the twofold role of reference diagrams able to i) present the data in an unambiguous manner (for a successful comparison) and ii) provide the guidelines for improving the interaction between rubber and cement paste. Results obtained pointed out the importance of i) correlating the crumb rubber size with the one of the relative mineral aggregate fraction that has been substituted, ii) premixing the solid elements for homogenizing the final composition, iii) pre-treating the rubber prior to use in the composite and iv) selecting a cement paste able to guarantee a reduction of the loss of mechanical properties in the final composite. Additionally, this study evidenced also how it is important to use a common language in science for a correct interpretation of the data. Therefore, in order to significantly reduce any misleading conclusions, authors hope that the approach here proposed will be extendedly adopted in the future.

## Figures and Tables

**Table 1:** Case studies selected for the data analysis.

Acronyms Adopted in the Present Study	Description (and Acronyms) from the Original Work	Original works	References
1A	Substitution of fine aggregate (SCRC30)	Yung et al. (2013)	[44]
1B	Substitution of fine aggregate (SCRC50)	Yung et al. (2013)	[44]
2A	Substitution of fine aggregate (F1)	Boudaoud et al. (2012)	[45]
2B	Substitution of coarse aggregate (F2)	Boudaoud et al. (2012)	[45]
3A	Substitution of fine aggregate, powder rubber	Kumar et al. (2014)	[46]
3B	Substitution of fine aggregate, powder and chipped rubber	Kumar et al. (2014)	[46]
4A	Substitution of fine aggregate	Issa et al. (2013)	[47]
5A	Substitution of fine aggregate (M7)	Youssf et al. (2016)	[48]
5B	Substitution of fine aggregate, rubber treated with NaOH at different time (M6, M8, M9)	Youssf et al. (2016)	[48]
6A	Substitution of fine aggregate (RC)	Lv et al. (2015)	[49]
7A	Substitution of fine aggregate (CF)	Liu et al. (2016)	[50]
7B	Substitution of both fine and coarse aggregates (CM)	Liu et al. (2016)	[50]
8A	Substitution of coarse aggregate, rubbers with different sizes (TC, CR, FCR, TC-CR, TC-FCR)	Gesoğlu et al. (2014)	[51]
9A	Substitution of fine aggregate (mix 16-20)	Mohammed et al. (2014)	[52]
9B	Substitution of fine aggregate (mix 21-25)	Mohammed et al. (2014)	[52]
10A	Substitution of coarse aggregate, ground rubber (GR-8)	Zheng et al. (2008)	[53]
10B	Substitution of coarse aggregate, crushed rubber (CR-40)	Zheng et al. (2008)	[53]
11A	Substitution of fine aggregate (RC)	Feng et al. (2018)	[54]
12A	Substitution of both fine and coarse aggregates (M25-R)	Tiwari et al. (2008)	[55]
13A	Substitution of fine aggregate (mix 2)	Gerges et al. (2018)	[56]
13B	Substitution of fine aggregate (mix 4)	Gerges et al. (2018)	[56]
14A	Substitution of fine aggregate (RC)	Liu et al (2013)	[58]
15A	Substitution of fine aggregate (series I)	Thomas et al (2016)	[59]
16A	Substitution of aggregate with fine rubber (FRC)	Topcu (1995)	[27]
16B	Substitution of aggregate with coarse rubber (CRC)	Topcu (1995)	[27]
17A	Substitution of fine aggregate (CR)	Kardos et al. (2015)	[60]
18A	Substitution of fine aggregate (RSCC)	Khalil et al. (2015)	[61]
19A	Substitution of fine aggregate (pre-treatment of rubber with NaOH) (CRC)	Li et al. (2018)	[62]
20A	Substitution of fine aggregate (RC-0.35)	Zhou et al (2018)	[63]
21A	Substitution of fine aggregate (CR)	Bisht et al. (2017)	[64]
22A	Substitution of fine aggregate (R)	Youssf et al. (2017)	[65]
23A	Substitution of fine aggregate (OH-)	Guo et al. (2017)	[66]
24A	Substitution of fine aggregate (RC-0.35)	Xue et al. (2018)	[67]
25A	Substitution of fine aggregate (01-RT)	Sgobba et al (2015)	[24]
25B	Substitution of fine aggregate (pre-treatment of rubber with NaOH) (01-RTL)	Sgobba et al (2015)	[24]
26A	Substitution of coarse aggregate (SCRC)	Aslani et al. (2018)	[37]
27A	Substitution of fine aggregate (FR)	Najim et al (2012)	[57]
27B	Substitution of coarse aggregate (CR)	Najim et al (2012)	[57]
27C	Substitution of both fine and coarse aggregates (FCR)	Najim et al (2012)	[57]

**Table 2:** Weight parameters adopted for the data analysis.

Components	Average Specific Weight for All the Data analyzed (kg m^−3^) ± St. Dev.
Rubber	1120 ± 64
Fine mineral aggregate	2649 ± 25
Coarse mineral aggregate	2672 ± 33
